# [Expression of Concern] MicroRNA-145-5p suppresses fascin to inhibit the invasion and migration of cervical carcinoma cells

**DOI:** 10.3892/mmr.2026.13988

**Published:** 2026-08-12

**Authors:** Shufang He, Guiyuan Yu, Ke Peng, Sisun Liu

Mol Med Rep 22: 5282–5292, 2020; DOI: 10.3892/mmr.2020.11592

Following the publication of this paper, it was drawn to the Editor's attention by a concerned reader that the Transwell migration and invasion assay experiments shown in Figs. 3D, 6D and F, and 7G and I contained three sets of overlapping sections of data, such that data which were intended to show the results of differently performed experiments had apparently been derived from the same original sources. In addition, the ‘si-FSCN1’ data panel for the migration assay experiments shown in Fig. 6D apparently reappeared in Fig. 6 of another paper written by different authors at different research institutes, which was submitted at a later date to the journal *Medical Science Monitor*.

The authors have been contacted by the Editorial Office to offer an explanation for the apparent anomalies in the presentation of the data in this paper, and we are awaiting their response. Owing to the fact that the Editorial Office has been made aware of potential issues surrounding the scientific integrity of this paper, we are issuing an Expression of Concern to notify readers of this potential problem while the Editorial Office continues to investigate this matter further.

